# Real-time Precise Point Positioning with a Xiaomi MI 8 Android Smartphone

**DOI:** 10.3390/s19122835

**Published:** 2019-06-25

**Authors:** Bo Chen, Chengfa Gao, Yongsheng Liu, Puyu Sun

**Affiliations:** School of Transportation, Southeast University, Nanjing 211189, China; 220172986@seu.edu.cn (B.C.); 220182986@seu.edu.cn (Y.L.); 220182988@seu.edu.cn (P.S.)

**Keywords:** Android smartphone, GNSS raw observations, Xiaomi MI 8, Real-time Precise Point Positioning

## Abstract

The Global Navigation Satellite System (GNSS) positioning technology using smartphones can be applied to many aspects of mass life, and the world’s first dual-frequency GNSS smartphone Xiaomi MI 8 represents a new trend in the development of GNSS positioning technology with mobile phones. The main purpose of this work is to explore the best real-time positioning performance that can be achieved on a smartphone without reference stations. By analyzing the GNSS raw measurements, it is found that all the three mobile phones tested have the phenomenon that the differences between pseudorange observations and carrier phase observations are not fixed, thus a PPP (precise point positioning) method is modified accordingly. Using a Xiaomi MI 8 smartphone, the modified real-time PPP positioning strategy which estimates two clock biases of smartphone was applied. The results show that using multi-GNSS systems data can effectively improve positioning performance; the average horizontal and vertical RMS positioning error are 0.81 and 1.65 m respectively (using GPS, BDS, and Galileo data); and the time required for each time period positioning errors in N and E directions to be under 1 m is less than 30s.

## 1. Introduction

Smartphones are an indispensable tool in people’s lives today, and the GNSS services provided by smartphones have greatly improved modern human life. In the development of GNSS navigation and positioning technology, accuracy has always been a key issue that restricts its further application to human production and life, and it is also suitable for the GNSS navigation and positioning on smartphones. 

In May 2016, Google provided an interface for accessing GNSS raw observations on the mobile devices with Android N operating system. Since then, researchers have begun to evaluate the quality of GNSS raw observations collected by mobile terminals and analyze the positioning performance. However, compared to the geodetic receivers, the assessment of smartphone GNSS raw observations and the analysis of smartphone positioning performance are still lacking.

Soon after, Google [1] released an app named GnssLogger which can easily capture and record GNSS observations. The source code of this app was also made open to the public. This app can save GNSS raw observations as text files, which greatly facilitates scholars’ research in this area all over the world. The next year, the European GNSS agency [2] released a white paper about using GNSS raw measurements on Android devices. This white paper not only shows the current research status in this respect, but also provides developers with detailed development instructions.

Early GNSS positioning experiments on smart devices mostly use the Nexus 9 tablet. The experiment of Gim et al. [3] is a pseudorange positioning test using the Nexus 9 tablet, and the result shows that the RMS positioning errors in horizontal and three-dimensional were 3.05 and 3.82 m. Realini et al. [4] carried out a single-frequency carrier phase double-difference positioning experiment using the tablet and several base stations, and the positioning accuracy better than 20 cm was achieved within 20 minutes. Zhang et al. [5] also used a Nexus 9 tablet for GNSS raw observations quality analysis and positioning test. The results show that the carrier-to-noise density ratio (C/N0) value of GNSS raw observations collected by mobile devices is 10 dB-Hz lower than the representative values obtained from a geodetic-quality antenna and receiver. Adopt TD (time-differenced) filtering method, achieved static positioning with horizontal and vertical RMS positioning errors less than 0.6 and 1.4 m, respectively. These studies have important implications for subsequent experiments using smartphones, however, the positioning of ordinary smartphones is not comparable to this tablet. 

In September 2017, Sikirica et al. [6] performed a pseudorange point positioning test under a good observation environment with a Huawei P10 smartphone, and the RMS positioning errors were about 10 m in the N and E directions and about 20 m in the U direction. In November 2017 Dabove et al. [7] conducted a NRKT (network real-time kinematic) positioning test using a Samsung S8+ smartphone and a Huawei P10 plus smartphone. The best positioning performance is the average horizontal positioning error of about 60 cm. In addition, Specht et al. [8] used six Samsung GALAXY smartphones for maritime differential positioning test. The results show that the horizontal positioning accuracy below 10 m can be achieved, which meeting most of the maritime navigation accuracy requirements.

This situation was improved to some extent in May 2018, when Xiaomi launched the world’s first dual-frequency GNSS smartphone with the Broadcom BCM47755 chipset which is a dual-frequency (L1/E1+L5/E5) GNSS chip [9]. This experiment shows that the GNSS raw measurements quality of each frequency is better than ordinary single-frequency smartphones (which is reported in Section 5).

The NSL’s FLAMINGO (Nottingham Scientific Limited’s fulfilling enhanced location accuracy in the mass-market through Initial Galileo Services) Team [10,11] assessed the quality of the GNSS raw measurements from Xiaomi MI 8 smartphones in July 2018. They considered that the carrier phase was not affected by duty cycling, and the raw observations quality of L5/E5 frequency are better than L1/E1 frequency. Subsequently, the same team conducted further dynamic positioning experiments on Xiaomi 8 smartphones. The horizontal RMS positioning errors of 1.17 and 2.23 m was achieved using the RTK (real-time kinematic) and precise point positioning (PPP) methods, respectively. Robustelli et al. [12] also performed a carrier phase differential positioning test on a Xiaomi MI 8 smartphone (using single frequency data) and achieved horizontal RMS positioning errors of 1.02 and 1.95 m at low and high multipath sites. 

In December 2018, Wu et al. [13] collected a whole day’s observations with a Xiaomi MI 8 smartphone and conducted the PPP positioning test. The experiments were performed using dual-frequency data (ionosphere-free combination of code and carrier phase) and single-frequency data. The dual-frequency model RMS positioning errors of E, N, and U directions are 21.8, 4.1, and 11.0 cm, respectively, but the result shows that the time for three-dimensional positioning errors to converge to 1 m is as long as 102 min. For single-frequency PPP, the time required for positioning errors in N and E directions to be under 1 m is more than 100 min. At the same time, the orbit and clock files used in this experiment are WHU (Wuhan University) final product, which means that the positioning result cannot represent the real-time PPP positioning performance. 

According to this published research, before the advent of the Xiaomi MI 8 smartphone, the accuracy of mobile phones positioning was on the level of several meters [6,7,8]. After Xiaomi MI 8 entered the market, its excellent GNSS raw observations quality quickly gained people’s attention, many scholars used it to conduct positioning tests. Research shows that the RTK and PPP positioning methods can effectively improve its positioning performance. Using a static RTK method, it is possible to obtain a horizontal positioning accuracy of approximately 1 m [11,12]. The experiment by Wu et al. shows that using the dual-frequency PPP method, after a long-time measurement, the positioning accuracy of the horizontal RMS less than 30 cm can be achieved [13]. The aim of their work is similar to ours: to obtain higher-precision positioning results on mobile phone without reference stations. But their study has some drawbacks: using the final orbit and clock errors products and cannot obtain high-precision positioning results in short time. This has resulted in their method not being able to be performed on the phone in real-time, which is obviously inconsistent with most mobile application scenarios. In addition, no one has made improvements in positioning strategy for mobile phones. Our experiment found that the differences between pseudorange observations and carrier phase observations of smartphones are not fixed (this phenomenon exists in all the three smartphones tested). We believe that it is necessary to modify the positioning strategy accordingly, and the experimental results verify our thought.

In this work, we use a single-frequency PPP strategy that estimates two clock biases of smartphone, a real-time high precision smartphone positioning is achieved. It should be noted that the definition of PPP (Precise Point Positioning) is a method for obtaining the absolute position of a single GNSS receiver using pseudorange, carrier phase observations, and high-precision IGS (International GNSS Service) products [14]. The accuracy of PPP should be centimeter-level, which is hard to achieve on a mobile phone until now. Because the method we used is same with PPP method, we also call it precise point positioning.

The experimental setup adopted is described in Section 2. The preliminary analysis helps us determine the specific positioning methodology are reported in Section 3. The PPP methodology followed to conduct our experiment are elaborated in Section 4. The experimental results are reported in Section 5. The conclusion and discussion of this work is in Section 6.

## 2. Experimental Setup

The main device used in this experiment is a Xiaomi MI 8 smartphone. For comparison analysis, a Huawei Honor 9 smartphone, a Huawei P10 smartphone, and two geodetic GNSS receivers (Hi-Target iRTK2) were also used. The Xiaomi 8 mobile phone is a dual-frequency GNSS smartphone that collects the first frequency band (L1/E1) signals of GPS (Global Positioning System), Galileo (Galileo satellite navigation system), BDS (BeiDou Navigation Satellite System), and GLONASS (GLObal NAvigation Satellite System), and the second frequency band (L5/E5a) signals of GPS and Galileo. Huawei P10 is a four-system single-frequency smartphone, and Huawei Honor 9 is a three-system (GPS, GLONASS, and BDS) single-frequency smartphone, and the geodetic receiver is a four-system three-frequency receiver.

**Figure 1:**
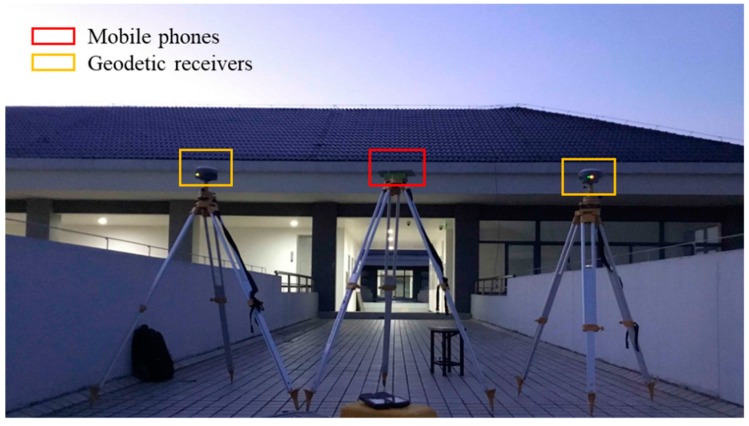
Mobile phones and geodetic receivers at the site of first and third dataset. There is a plastic board on the top of the middle tripod, and smartphones are placed on the board.

Three datasets were collected in total with a sampling rate of 1 s. The first and third datasets were in the same site: the top of the teaching building No. 5 in Southeast University Jiulonghu Campus, as shown in Figure 1. This site is in a low multipath environment. Since the precise coordinates of the site are unknown, these datasets are mainly used for the raw measurements’ quality analysis. The first dataset was collected on July 12, 2018, over a time span of about three hours. The devices used were a Huawei P10 smartphone, a Huawei Honor 9 smartphone, and two geodetic receivers. The third dataset was collected on October 29, 2018, over a time span about one hour, and the devices used are a Xiaomi MI 8 smartphone and a geodetic receiver.

**Figure 2:**
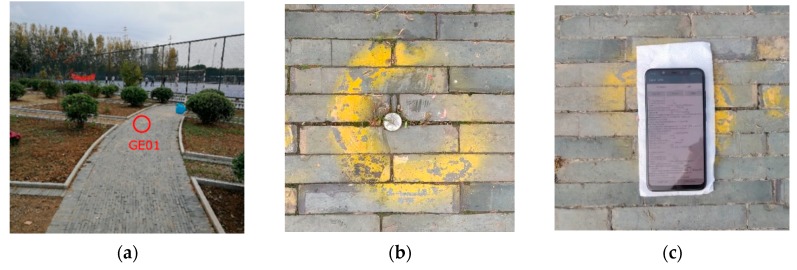
The control point named GE01 at Southeast University Jiulonghu Campus. The precise coordinates of this control point are obtained by a geodesic receiver through the network RTK positioning method. (**a**) Distant view of the control point; (**b**) Close-up of the control point; (**c**) A Xiaomi MI 8 smartphone placed on the control point.

The second dataset was collected on October 19, 2018, and the site is the GE01 control point in Southeast University Jiulonghu Campus, as shown in Figure 2. The device used is a Xiaomi MI 8 smartphone. Five time periods were observed, about 6 minutes each time. The precise coordinates of this site are known, and the observations are used for positioning tests.

Based on the Gnsslogger APP [1], we developed the GnssLiuYS APP for data logging, pre-processing and sending. Besides, two Windows desktop programs were developed to achieve converting the original data (which obtained from the GnssLiuYS APP) to the RINEX (Receiver Independent Exchange Format) format and PPP calculation. 

## 3. Preliminary Analysis

**Figure 3:**
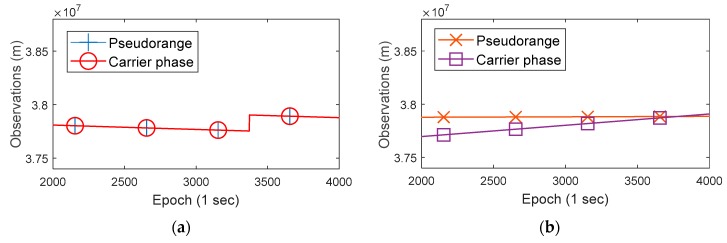
The pseudorange and carrier phase observations (BDS 02 satellite) of a geodetic receiver and a Huawei P10 smartphone. (**a**) The observations of a geodetic receiver; (**b**) The observations of a Huawei P10 smartphone. The actual acquired carrier phase of smartphones is a set of data that is cumulatively incremented from 0 m at the beginning of observation, thus the values are small. In this figure, the mobile phone carrier phase values are added a constant.

We conducted a preliminary analysis on the obtained GNSS raw observations and determined the specific positioning methodology accordingly. Figure 3 shows the GNSS raw observations (BDS 02 satellite) of a geodetic receiver and a Huawei P10 smartphone in the same time. 

In panel (a) of Figure 3, the blue and red lines are coincident (so only the red line is visible), which indicates that the pseudorange and carrier phase observations (in meters) of the geodetic receiver are consistent. In fact, the difference between the two values is equal to the carrier phase integer ambiguity in meters. However, in panel (b), the orange and purple lines are not consistent and have different slopes, which indicates that the differences between the pseudorange and the carrier phase observations of Huawei P10 are not fixed. At the same time, we found that this phenomenon also exists in Huawei Honor 9 and Xiaomi MI 8. This property is different from the geodetic receivers, which affects the use of carrier phase measurements of smartphone. By the way, the raw measurements of the geodetic receiver have a large jump after a period of time, which is caused by receiver’s clock jump and does not affect the positioning solutions [15].

**Figure 4:**
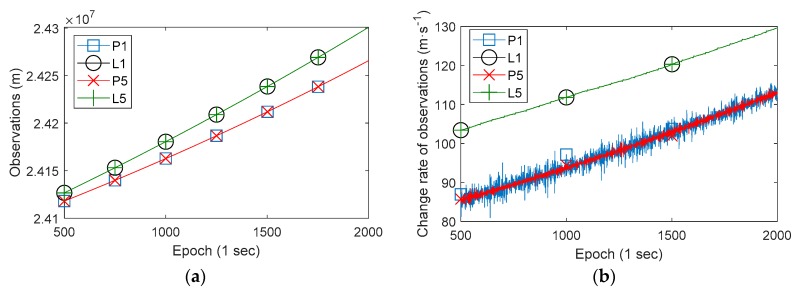
The observations (Galileo 03 satellite) and its change rate of a Xiaomi MI 8 smartphone. (**a**) The raw observations; (**b**) The change rate of observations.

Particularly for the Xiaomi MI 8 smartphone, the dual-frequency raw observations (Galileo 03 satellite) and its change rate are shown in Figure 4. In panel (a), P1 and P5 denote pseudorange observations of two frequencies E1 and E5, L1 and L5 denote corresponding carrier phase observations. In panel (b), P1, P5, L1, and L5 denote a corresponding change rate. Obviously, the pseudorange observations of two frequencies are coincident (the corresponding two lines are coincident), and the carrier phase observations of two frequencies are also coincident. However, it is evidently that the differences between pseudorange observations and carrier phase observations are not fixed, which is same with Huawei P10 and Huawei Honor 9. Panel (b) shows that the change rate of observations also exists the same phenomenon. At the same time, P5 is more stable than P1 according panel (b), which means the data quality of P5 is better obviously.

**Figure 5:**
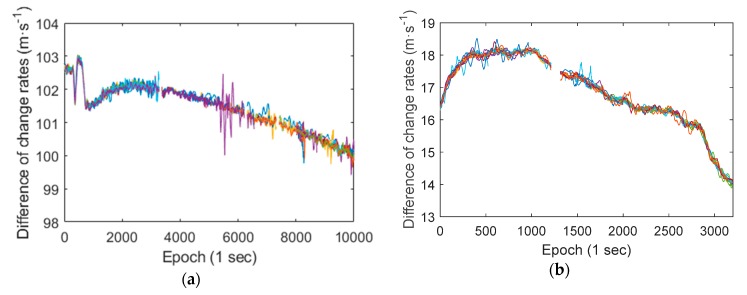
The differences between pseudorange change rate and carrier phase change rate of all GNSS satellites of a Huawei P10 smartphone and a Xiaomi MI 8 smartphone (L1/E1 frequency). (**a**) Huawei P10 smartphone; (**b**) Xiaomi MI 8 smartphone (L1/E1 frequency).

As shown in Figure 4, the differences between the smartphone pseudorange and carrier phase observations are gradually increased, and the difference values are large. At the same time, the differences between pseudorange change rate and carrier phase change rate are relatively stable. Therefore, in order to better evaluate this phenomenon in different satellites’ observations, we calculated the differences between pseudorange change rate and carrier phase (in meters) change rate of all GNSS satellites of a Huawei P10 smartphone and a Xiaomi MI 8 smartphone (L1/E1 frequency), as shown in Figure 5. The RMS of smartphone pseudorange observations exceeds a few meters, thus the difference value between the change rate of pseudorange and the change rate of carrier phase observations should have an amplitude of several meters too. This makes it difficult to assess the degree of agreement between different satellites observations, thus the difference values are calculated with a window of 100 epochs (take the average value of 100 epochs). 

There are a total of 26 lines in panel (a) of Figure 5, and a total of 23 lines in panel (b), the different colored lines represent different satellites. Although there are some deviations, most of the lines are coincident, and the lines of Xiaomi MI 8 are more coincident. Which indicates that the differences between the change rate of pseudorange observations and the change rate of carrier phase observations of all satellites are consistent. It also can be seen that the differences of change rate are slowly changing during the observation period, and the variation range is about several meters within one hour.

Since the influence of the device clock bias on all satellites observations is the same, we believe that estimating two clock biases in positioning process can effectively weaken the impact of the phenomenon that the differences between the pseudorange and the carrier phase observations of mobile phone are not fixed.

## 4. Methodology

During the first and third dataset collections, mobile phones and geodetic GNSS receivers were synchronized at the same location. The accuracy of the carrier phase observation of geodetic receivers is much better than the pseudorange observations of smartphones. We took the carrier phase observations of the geodetic receivers as the standard value and calculated the RMS of pseudorange observations of smartphones. The specific steps are as follow:

1. Calculating the difference values between the mobile phone’s pseudorange observations and the same time geodetic receiver’s carrier phase observations (in meters);
2. Performing linear regression (to eliminate systematic deviation such as the influence of geodetic receiver clock bias) on the difference values within a certain time window (100 epochs), obtaining the regression residuals;
3. Calculating the RMS value of the regression residuals, that is the RMS of smartphone’s pseudorange observations.

For the carrier phase observations, by calculating the RMS of carrier phase observations change rate of each device (also with the window of 100 epochs, and the linear regression was also performed), the qualities of carrier phase observations of tested devices are compared and analyzed. The aim of analyzing the quality of mobile phones’ GNSS raw observations is to help us determine the zenith direction variance of GNSS observations in Kalman filter process.

According to the preliminary analysis, there are two clock biases of smartphone that need to be estimated in the positioning process. Taking the GPS as an example, the observation equations can be described by:

(1) $$P_{i}^{g}=\rho_{i}^{g}+cd{\tilde{t}}_{P}-cdT^{g}+d_{orb}^{g}+d_{trop}^{g}+d_{ion}^{g}+\epsilon_{P}^{g},$$

(2) $$\Phi_{i}^{g}=\rho_{i}^{g}+cd{\tilde{t}}_{\Phi }-cdT^{g}+d_{orb}^{g}+d_{trop}^{g}-d_{ion}^{g}+{\tilde{N}}_{i}^{g}+\epsilon_{\Phi }^{g},$$

where the superscript g denotes GPS, the subscript i denotes the i-th satellite, $P_{i}^{g}$ and $\Phi_{i}^{g}$ are the pseudorange observation and carrier phase observation in meters, $\rho_{i}^{g}$ is the distance between the mobile phone and the satellite, c is the speed of light, $d{\tilde{t}}_{P}$ and $d{\tilde{t}}_{\Phi }$ are the clock biases of pseudorange observation and carrier phase observation, $dT^{g}$ is the clock bias of satellite, $d_{orb}^{g}$ is the orbit error of satellite, $d_{trop}^{g}$ is the tropospheric delay, $d_{ion}^{g}$ is the ionospheric delay, ${\tilde{N}}_{i}^{g}$ is the integer ambiguity of carrier phase in meters, and $\epsilon_{\Phi }^{g}$ the is residual error.

There are too few Galileo satellites and GPS satellites with L5 signals observed by mobile phones, however, the parameters to be estimated are too many, and thus the PPP positioning model used is a single-frequency non-difference model.

Using precise ephemeris and precise clock bias files to reduce the orbital errors and satellite clock biases, weakening the ionospheric delay error with corresponding product, the observation equation was simplified as in Reference [16]:

(3) $$P_{i}^{g}=\rho_{i}^{g}+cd{\tilde{t}}_{P}+d_{trop}^{g}+\epsilon_{P}^{g},$$

(4) $$\Phi_{i}^{g}=\rho_{i}^{g}+cd{\tilde{t}}_{\Phi }+d_{trop}^{g}+{\tilde{N}}_{i}^{g}+\epsilon_{\Phi }^{g},$$

(5) $$P_{j}^{c}=\rho_{j}^{c}+cd{\tilde{t}}_{P}+cd{\tilde{t}}_{sys}^{c}+d_{trop}^{c}+\epsilon_{P}^{c},$$

(6) $$\Phi_{j}^{c}=\rho_{j}^{c}+cd{\tilde{t}}_{\Phi }+cd{\tilde{t}}_{sys}^{c}+d_{trop}^{c}+{\tilde{N}}_{k}^{c}+\epsilon_{\Phi }^{c},$$

where the superscript c denotes BDS, the subscript j denotes the j-th satellite, $d{\tilde{t}}_{sys}^{c}$ is the time bias between BDS and GPS, and the other parameters have the same meaning as Equations (1) and (2). In addition, Galileo and GLONASS systems also have corresponding observation equations, and their main difference is the $d{\tilde{t}}_{sys}$.

Suppose that $n_{1}$ GPS satellites, $n_{2}$ BDS satellites are observed at a certain time. The vector of parameters to be estimated is:

(7) $$X^{T}=\left[x\;y\;z\;cd{\tilde{t}}_{P}\;\;cd{\tilde{t}}_{\Phi }\;\;cd{\tilde{t}}_{sys}^{c}\;d_{trop}\;{\tilde{N}}_{1}^{g}\;\;\cdots \;{\tilde{N}}_{n_{1}}^{g}\;{\tilde{N}}_{n_{1}+1}^{c}\;\;\cdots \;{\tilde{N}}_{n_{1}+n_{2}}^{c}\right],$$

where the x, y, and z are the absolute position of the mobile phone. Therefore, the total number of observation equations is $2\times \left(n_{1}+n_{2}\right)$, the parameters to be estimated are $7+n_{1}+n_{2}$, and the number of redundant observations is $n_{1}+n_{2}-$ 7.

The parameters estimation method used is the standard static Kalman filter [16]. In particular, since the vector of parameters to be estimated is modified, the matrix of observation coefficients in the filtering process needs to be modified accordingly. When the observations equation is as shown in Equation (8), the coefficient matrix should be as shown in Equation (9).

(8) $$l^{T}=\left[P_{1}^{g}\;\;\Phi_{1}^{g}\;\cdots \;P_{n_{1}}^{g}\;\;\Phi_{n_{1}}^{g}\;\;P_{n_{1}+1}^{c}\;\Phi_{n_{1}+1}^{c}\;\cdots \;P_{n_{1}+n_{2}}^{c}\;\Phi_{n_{1}+n_{2}}^{c}\right],$$

(9) $$H=|\begin{array}{ccccccccccccc} \alpha_{1} & \beta_{1} & \gamma_{1} & 1 & 0 & 0 & MF_{1} & 0 & \cdots & 0 & 0 & \cdots & 0\\ \alpha_{1} & \beta_{1} & \gamma_{1} & 0 & 1 & 0 & MF_{1} & 1 & \cdots & 0 & 0 & \cdots & 0\\ \vdots & \vdots & \vdots & \vdots & \vdots & \vdots & \vdots & \vdots & \vdots & \vdots & \vdots & \vdots & \vdots \\ \alpha_{n_{1}} & \beta_{n_{1}} & \gamma_{n_{1}} & 1 & 0 & 0 & MF_{n_{1}} & 0 & \cdots & 0 & 0 & \cdots & 0\\ \alpha_{n_{1}} & \beta_{n_{1}} & \gamma_{n_{1}} & 0 & 1 & 0 & MF_{n_{1}} & 0 & \cdots & 1 & 0 & \cdots & 0\\ \alpha_{n_{1}+1} & \beta_{n_{1}+1} & \gamma_{n_{1}+1} & 1 & 0 & 1 & MF_{n_{1}+1} & 0 & \cdots & 0 & 0 & \cdots & 0\\ \alpha_{n_{1}+1} & \beta_{n_{1}+1} & \gamma_{n_{1}+1} & 0 & 1 & 1 & MF_{n_{1}+1} & 0 & \cdots & 0 & 1 & \cdots & 0\\ \vdots & \vdots & \vdots & \vdots & \vdots & \vdots & \vdots & \vdots & \vdots & \vdots & \vdots & \vdots & \vdots \\ \alpha_{n_{1}+n_{2}} & \beta_{n_{1}+n_{2}} & \gamma_{n_{1}+n_{2}} & 1 & 0 & 1 & MF_{n_{1}+n_{2}} & 0 & \cdots & 0 & 0 & \cdots & 0\\ \alpha_{n_{1}+n_{2}} & \beta_{n_{1}+n_{2}} & \gamma_{n_{1}+n_{2}} & 0 & 1 & 1 & MF_{n_{1}+n_{2}} & 0 & \cdots & 0 & 0 & \cdots & 1 \end{array}|,$$

where the α, β, and γ are the direction cosines from mobile phone to satellite; MF is the tropospheric projection coefficient, and the projection equation used is the Neill model [17]. The Equations (7)–(9) are suitable when using GPS and BDS data in positioning process, which need to be modified when using other GNSS systems data.

The specific PPP positioning settings are shown in Table 1 below.

The satellite orbit, clock bias and ionospheric delay products mentioned in Table 1 were downloaded from the IGS Data Center of Wuhan University (http://www.igs.gnsswhu.cn/). Specifically, the orbit and clock bias are mitigated by WHU (Wuhan University) ultra-rapid products predicted part, the ionospheric delay is mitigated by CODE (The Center for Orbit Determination in Europe) 2-day predicted GIM (global ionosphere maps). Previous studies show that using the final GIM products can correct approximately 80% of the ionospheric delay, but the residual ionospheric delay error still has a decimeter level harmful effect on the positioning results of single-frequency PPP [20]. Moreover, the predicted GIM product used in this study has a worse correction effect than the final product, which is an important factor affecting the performance of our positioning experiments. All the IGS data products used were predicted products, indicating that the method used in this work is suitable for real-time positioning.

## 5. Results

Using the method described in Section 3, calculating the RMS of pseudorange observations of each mobile phone, the results are shown in Table 2 below.

Table 3 shows each device’s RMS of carrier phase observation change rates. The average RMS of carrier phase observation change rates of Huawei Honor 9, Huawei P10, and Xiaomi MI 8 mobile phone are 5.5, 6.4 and 4.0 times of the geodetic receiver, respectively. Table 2 and Table 3 show that the quality of the raw observations of Xiaomi MI 8 is obviously improved compare with single-frequency mobile phones.

In the three data collection processes, the number of GPS, BDS, and GLONASS satellites that can be observed by smartphones is relatively stable, 6-8 for GPS, 6-7 for BDS; but the number of Galileo satellites observed is small, 3 for Huawei P10 and 0 for Huawei Honor 9, while the Xiaomi MI 8 observed 3 in the third dataset collection process, only 1 in the second dataset collection process. In addition, the observed GPS satellites with L5 signal are 2-3. Too few observed satellites with L5/E5 signals has resulted in inefficient use of L5/E5 frequency data, and thus positioning tests in this work are all use single frequency observations.

Using the PPP method detailed in Section 3, positioning tests was performed with a Xiaomi 8 smartphone. In order to evaluate the impact of using different constellation combinations on the positioning results, we used a variety of GNSS systems combinations for testing. The RMS positioning errors of representative GNSS system combinations are shown in Table 4. In general, as the number of constellations used increases, the positioning performance gradually improves. There are 6 parameters that need to be estimated in our positioning strategy (when using single GNSS system data), and the number of satellites observed of GPS/BDS systems is 6-8, which results in little or no redundant observations. Thus, the performance of single constellation positioning is poor. Thanks to the number of observable GPS and BDS satellites, the result using GPS and BDS data is obviously better than using single constellation data. Since the number of Galileo satellites observed by mobile phones is small, the positioning performance is not obviously improved after adding Galileo data. For GLONASS, after adding the its data, the positioning performance of some time periods can be improved, and some time periods (the 1st and 5th time periods) are badly affected. The average horizontal RMS positioning error is 0.81m, and the average vertical (up direction) RMS positioning error is 1.65m (using GPS, BDS, and Galileo data).

**Figure 6:**
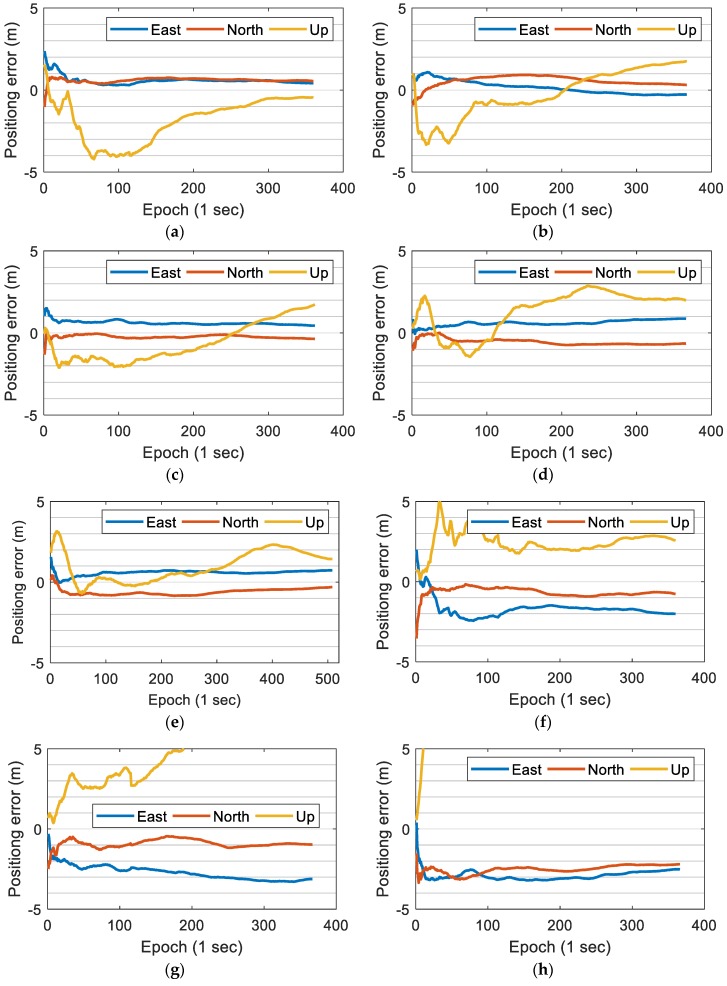
The North (N), East (E), and Up (U) direction errors of precise point positioning (PPP) positioning results of the five time periods. (**a**) 1st time period (G + C + E); (**b**) 2nd time period (G + C + E); (**c**) 3rd time period (G + C + E); (**d**) 4th time period (G + C + E); (**e**) 5th time period (G + C + E); (**f**) 1st time period (GPS); (**g**) 2nd time period (GPS); (**h**) 4th time period (GPS). The “G”, “C” and “E” denote GPS, BDS, and Galileo.

The specific positioning error curves are shown in Figure 6 (panel (a)–(e) for using GPS, BDS, and Galileo data, panel (f)–(h) for using GPS data only). When using GPS, BDS, and Galileo data, after observing for a section of time, the East and North direction errors of all the five time periods can converge to less than 1 m and achieve a relatively stable positioning result; but the U direction positioning result is not stable enough. When using GPS data only, the positioning results are more unstable, and the systematic errors are also greater. The loss of positioning accuracy in the U direction is more obvious compared to the E and N directions. We believe that there are two reasons for the poor performance of single system positioning: the number of satellites observed by mobile phones is not enough, and the quality of smartphone raw GNSS observations is poor. This is consistent with the idea of surveying adjustment: when the accuracy of a single measurement is poor, a large number of repeated observations are needed to improve the overall accuracy. If the number of available satellites continues to increase, the positioning performance may be further improved. However, the GLONASS data of mobile phone seems to contain more gross errors than other GNSS systems, and sometimes it has a bad influence on the positioning results, so we tend to abandon GLONASS data if there are enough visible satellites. 

The accuracy of 1 m can meet the needs of most non-professional fields, even some professional fields with low precision requirements [13]. The time required for positioning errors in N and E to both be less than 1 m and the subsequent epoch error to no longer exceed 1 m is counted, as shown in Table 5. The time required for positioning errors in N and E directions to be under 1 m of each time period is less than 30 s, which indicates that the modified PPP strategy can be applied to real-time positioning and provides a slightly delayed high-precision positioning result. The positioning performance achieved in this work is the best real-time positioning performance that can be achieved with ordinary smartphones until now.

We also performed positioning tests using the ordinary PPP strategy which estimates single device time bias and the positioning errors are of the level of several meters. As reported by Wu et al., using single-frequency GNSS data, the time required for positioning errors in N and E directions to be under 1 m is more than 100 min, even when using the widely used software RTKlib for processing [13]. This indicates that evaluating two clock bias for the Xiaomi MI 8 smartphone is valid.

## 6. Conclusion and Discussion

In this paper, we compared and analyzed the quality of GNSS raw observations of different smartphones. Using a modified single-frequency PPP strategy, a real-time high precision smartphone positioning is achieved.

Taking the geodesic receivers’ carrier phase observations as the standard values, the average RMS of the pseudorange observations of Huawei Honor 9, Huawei P10, and Xiaomi MI 8 smartphones are 12.23 m, 8.58 m, and 3.97 m, respectively. The average RMS of the carrier phase observation change rates of these three smartphones are 5.5, 6.4, and 4.0 times of the geodetic receivers, respectively. As the world’s first dual-frequency GNSS smartphone, the quality of the GNSS raw observations of the Xiaomi MI 8 smartphone is greatly improved compared to the ordinary single-frequency smartphones. In the L1/E1 band, the Xiaomi MI 8 smartphone has smoother pseudorange and carrier observations, and meanwhile the raw observations of the L5/E5 band are even better. 

By analyzing the GNSS raw observations, it is found that all the three smartphones tested have the phenomenon that the differences between pseudorange observations and carrier phase observations are not fixed. This may be a general problem with smartphones that needs to be modified in the positioning algorithm. Using the real-time single-frequency PPP positioning strategy which estimated double clock biases of smartphone, the results show that using more GNSS systems data can effectively improve positioning performance, but GLONASS data sometimes have a bad effect on positioning performance. In the case of using GPS, BDS, and Galileo data, the horizontal and vertical RMS positioning error of 0.81 and 1.65 m on average are achieved with a Xiaomi MI 8 smartphone. The time required for positioning errors in N and E directions to be under 1 m of each time period is less than 30 s, which is the best real-time positioning performance that can be achieved with smartphones.

Compared with the experimental results of Wu et al. [13] who conducted similar studies, their positioning accuracy is better than our work (the average horizontal RMS positioning error after convergence less than 0.30 m). However, because we used single-frequency data, the ionospheric errors could not be effectively eliminated, and the predicted IGS products used were predicted products (for real-time positioning), so the difference in accuracy is acceptable. More importantly, the time required for high positioning accuracy of our work is much less, which means that our work is more in line with the actual application scenarios of smart phones.

Unfortunately, due to the small number of Galileo satellites and GPS satellites with L5 signals observed, we have not been able to effectively use L5/E5 frequency data. Previous studies show that the L5/E5 frequency data quality is significantly better than L1/E1 [11,12,13], and the combination of L1/E1 and L5/E5 can effectively eliminate the ionospheric delay error. How to effectively use L5/E5 frequency data will be a focus of our next work. Besides this, differential positioning, especially RTK positioning, can also effectively improve smartphone positioning performance [11,12]. For this method, the influence on positioning results caused by the phenomenon we found are not clear, which needs further analyses and discussions.

We are trying to develop a positioning APP that uses the double clock bias PPP strategy. The preliminary results show that the method has practical effects, and for ordinary single frequency smartphones, their positioning performance can be improved too. A mobile phone positioning app with high precision can effectively improve the user experience of public and may be applied to some professional work with low precision requirements, such as external annotation and cadastral survey. However, it can be foreseen that the GNSS chip in the mobile phone is developing rapidly and the updates of Android operating system will also affect the quality of smartphone raw GNSS observations. Whether the future smartphones have the phenomenon that the differences of pseudorange and carrier phase observations are not fixed needs to be determined according to specific analysis. 

## Figures and Tables

**Table 1:** The detailed settings of the precise point positioning (PPP) method used.

Setting Items	Details
Observations used	Single-frequency pseudorange and carrier phase
Satellite orbit errors and clock biases	Precise predicting orbit and clock bias files
Ionospheric delay errors	Predicting ionospheric grid files
Tropospheric delay errors	Correcting with the Hopfield model [16,18] and the remainder is estimated as a parameter
Effects of relativity and earth rotation	Correcting with the corresponding formula [19]
Weighting method	Satellite elevation angle
Integer ambiguities of carrier phase	Estimating float solution
Cutoff satellite elevation angle	10°
Parameters estimation method	Standard static Kalman filter

**Table 2:** The RMS of pseudorange observations of each mobile phone with different global navigation satellite systems (GNSS), where P1 and P5 denote pseudorange observations of two frequencies L1/E1 and L5/E5.

Devices	RMS of Pseudorange Observations (m)						
	Average	GPS P1	GPS P5	Galileo P1	Galileo P5	BDS	GLONASS
Huawei Honor 9	12.23	10.96	\	\	\	10.96	14.83
Huawei P10	8.58	8.11	\	7.39	\	8.11	12.57
Xiaomi MI 8	3.97	3.59	2.05	2.92	1.78	3.26	8.28

**Table 3:** The RMS of carrier phase observation change rates of each mobile phone with different global navigation satellite system (GNSS) systems, where L1 and L5 denote carrier phase observations of two frequencies L1/E1 and L5/E5.

Devices	RMS of Carrier Phase Observation Change Rates (m/s)						
	Average	GPS L1	GPS L5	Galileo L1	Galileo L5	BDS	GLONASS
iRTK2 (Geodetic receiver)	0.021	0.021	\	0.021	\	0.021	0.022
Huawei Honor 9	0.115	0.098	\	\	\	0.071	0.143
Huawei P10	0.135	0.134	\	0.010	\	0.143	0.137
Xiaomi MI 8	0.083	0.068	0.073	0.067	0.114	0.092	0.091

**Table 4:** The RMS positioning errors of the five time periods using different global navigation satellite systems (GNSS) system combinations. The “G”, “C”, “E” and “R” denote GPS, BDS, Galileo and GLONASS. The “U” and “H” denote “up” and “horizontal”.

Time periods	RMS Positioning Errors (m)									
	GPS		BDS		G + C		G + C + E		G + C + E + R	
	U	H	U	H	U	H	U	H	U	H
1st	2.56	1.92	7.77	3.28	2.19	0.89	2.24	0.89	2.28	1.05
2nd	5.25	2.92	2.41	3.94	1.65	0.77	1.47	0.76	1.39	0.56
3rd	5.33	2.84	6.59	4.11	1.37	0.67	1.33	0.68	1.39	0.72
4th	10.54	3.86	9.69	4.80	1.99	0.85	1.86	0.85	1.16	0.79
5th	6.15	3.54	18.77	5.78	1.65	0.87	1.35	0.88	2.14	0.76
Average	5.97	3.02	9.05	4.38	1.77	0.81	1.65	0.81	1.67	0.78

**Table 5:** The time required for positioning errors in N and E directions less than 1 m of all time periods.

Time Periods	Time Required for Positioning Errors in N and E Directions to Be Less than 1 m (s)
1st	26
2nd	25
3rd	11
4th	2
5th	3
